# Education Research: Secondary Trauma in Third-Year Medical Students During a Neurology/Emergency Medicine Clerkship

**DOI:** 10.1212/NE9.0000000000200244

**Published:** 2025-09-08

**Authors:** Sydney L. Short, Saadia Akhtar, Mallory Johnson, Lillian R. Sims, Larry B. Goldstein, Kimberly S. Jones

**Affiliations:** 1 College of Medicine, University of Kentucky;; 2 Department of Behavioral Science, University of Kentucky; and; 3 Department of Neurology, University of Kentucky.

## Abstract

**Background and Objectives:**

Exposure to emotionally taxing patients can lead to secondary trauma that can result in burnout and emotional exhaustion in healthcare providers. Understanding whether secondary trauma is present in medical students could provide an opportunity to address the issue earlier in physician training. We sought to determine if secondary trauma characteristics exist in third-year medical students during a combined neurology/emergency medicine clerkship.

**Methods:**

Six cohorts of third-year medical students at a MD-granting institution were invited to complete the Professional Quality of Life Survey, a widely used tool for assessing secondary trauma and its associated symptoms of compassion fatigue and burnout in healthcare workers. This was combined with supplemental questions and a standard postrotation survey to explore medical students' emotional experiences during the clerkship. The strength of the associations was measured with Cramér's V.

**Results:**

The survey had a 51% response rate (n = 100) with 11.3% reporting symptoms of secondary trauma. Of the respondents, 81% identified as White, 6% as Black/African American, 6% as Asian, and 2% as American Arab/Middle Eastern/North African; 60% identified as female and 37% as male. Contributors to secondary trauma could be grouped into 4 themes: 1, feeling helpless in the student role; 2, witnessing the physical consequences of patient diseases; 3, personal trauma history; and 4, empathy burden. Most students expected the level of emotional distress they experienced during the clerkship. Hobbies, wellness activities, talking to others, and avoidance were common reported coping strategies. Participation in hobbies was associated with less emotional exhaustion (Cramér's V = 0.372; *p* = 0.001). Feelings of increased preparedness were associated with less emotional exhaustion (Cramér's V = 0.395; *p* = 0.005).

**Discussion:**

Secondary trauma characteristics were identified in 11.3% of third-year medical students during a combined neurology/emergency medicine clerkship. This provides an opportunity to incorporate interventions that are effective in reducing secondary trauma characteristics in physicians during this early stage of training. Recognizing and proactively reducing secondary trauma during the clerkship experience may have longer-term benefits during later stages of medical training and practice.

## Introduction

Secondary trauma occurs when an individual experiences emotional distress from indirect exposure to the traumatic experiences of others. Terms including secondary traumatic stress, vicarious trauma, and second victim have been used interchangeably with secondary trauma to describe this emotional experience.^1^ Healthcare providers are at increased risk of developing secondary trauma due to exposure to patients' current and prior traumas, whether by actively treating the direct consequences of a traumatic event or listening to patients' vivid descriptions of these events during the history taking process.^1^ Secondary trauma is an umbrella term and can be reflected in a variety of specific symptoms including compassion fatigue, emotional exhaustion, burnout, and reduced compassion satisfaction and occurs in both resident and attending physicians.^1^

The risk of exposure to secondary trauma differs across healthcare contexts with some specialties carrying more risk due to the nature of their patient populations. In one study, neurologists had the second highest percentage of secondary trauma among 29 specialties, and emotional burden is found in clinical neurologists who diagnose patients with chronic neurologic illnesses.^1,2^ There are various aspects of neurologists' duties that likely contribute to the secondary trauma observed in this population, including diagnosing acute and chronic conditions that may have high morbidity and mortality, performing brain death examinations, and treating victims of abusive head trauma. Additional factors that may heighten symptoms of secondary trauma in neurologists include inadequate compensation for time spent performing nonprocedural tasks, inability to spend appropriate time with patients due to reduced workforce and increasing patient populations, and working more hours on average compared with other physicians in the United States.^3,4^ Medical students are exposed to these clinical and structural obstacles faced by neurologists during their clerkship, which likely affects their emotional experiences.

The detrimental impact of exposure to distressing patients occurs at all stages of neurology training.^5^ Medical students are exposed to a wide variety of clinical experiences, with a rapid increase in patient encounters during the clerkship stage of training, typically in the third year of the medical school curriculum. These students face unique challenges, including being required to rotate with specialties that involve exposure to trauma even if they do not plan to practice in that environment after graduation. They also spend only limited time with specialty-specific care teams, giving them even less opportunity to debrief after experiencing intense situations such as rounding in the intensive care unit, hearing about patients' traumatic experiences, and witnessing life-altering conversations with patients' loved ones. These trainees are at risk for burnout, secondary trauma, and decreased compassion satisfaction.^6^ One study found that secondary trauma symptoms increase throughout the academic year.^7^ During their neurology experience, trainees are involved in the care of hospitalized patients who may have devastating conditions. Interventions including counseling, compassionate connections with patients, and optimal work-life balance can prevent or alleviate secondary trauma symptoms in physicians.^5,8,9^ Information on the effects of participation in neurology rotations and whether it is associated with secondary trauma symptoms in medical students is pivotal to understanding if implementing these strategies in the medical student population during their neurology clerkship could address these deleterious effects and decrease potential negative views of the specialty.

Although the presence of secondary trauma in practicing physicians is known, research on secondary trauma in medical education is scarce. Understanding when symptoms of secondary trauma arise in medical trainees and determining which components of medical education put students at highest risk are critical to understanding how to best combat this issue. To help bridge this gap in knowledge in medical education, we focused on the relationship between a combined neurology/emergency medicine clerkship and secondary trauma. We hypothesized that secondary trauma risk to medical students is also increased during their experience on a neurology/emergency medicine clerkship. The goal of this study was to determine the presence of secondary trauma in addition to associated symptoms of emotional exhaustion, decreased compassion satisfaction, and burnout among medical students following a combined neurology/emergency medicine clerkship.

## Methods

### Study Population

The study population consisted of 6 cohorts of third-year medical students from a MD-granting College of Medicine from the institution's primary and 3 regional campuses. The curriculum includes a mandatory, 2-week neurology clerkship that is combined with a 4-week emergency medicine clerkship. The neurology component can be the first, second, or third 2-week block of the experience. During the neurology rotation, students are exposed to various aspects of the specialty while working closely with resident and attending physicians. The clerkship includes 1 week on stroke units (progressive or intensive care units) and 1 week on the general or child neurology inpatient service with afternoon outpatient clinics. The college's Office of Medical Education assigns clerkship schedules based on student preferences.

### Survey

In addition to a standard postclerkship questionnaire, all students were asked to complete an optional online Qualtrics survey at the end of their combined neurology/emergency medicine 6-week clerkship during the 2023–2024 academic year that had to be completed within 1 week. The survey was intended to collect qualitative and quantitative data reflecting their overall emotional experiences during the 2 rotations regardless of the timing of the neurology component. It was not possible to separately assess the neurology and emergency medicine portions of the clerkship. Questions focused on emotional trauma history, feelings, and causes of emotional exhaustion, coping strategies, and curricular changes they felt would be helpful for future third-year students (eAppendix 1). Open response options were included to allow students to expand on their thoughts and experiences.

Students were also invited to complete the Professional Quality of Life Survey (ProQOL) Version 5, which comprised of 30 questions assessing compassion satisfaction, burnout, and secondary trauma among healthcare workers over the prior 30 days^10^ The ProQOL is the most commonly used tool to assess the effects of working with individuals exposed to trauma and has been found to have appropriate construct validity.^10^ It uses a standardized questionnaire that has been used to evaluate compassion satisfaction and secondary trauma itself as well as symptoms often associated with secondary trauma burnout and compassion fatigue in healthcare providers from a variety of specialties.^11,12^ The ProQOL Version 5 is available in multiple languages and has been used to assess the presence of these issues in medical students in countries such as Taiwan.^13^ Medical schools often also conduct end-of-course evaluations to gather student feedback on their experiences during the course including resources, structure, and content that can be used to make effective curriculum changes.^14^

Students were compensated ($10) for participation. Students' email addresses were collected to determine who had completed the survey, but these email addresses were not linked to answers to protect confidentiality. This allowed for the students who completed the survey to receive compensation and prevented a student from submitting more than one survey. Survey authors reviewed the questionnaire on their personal devices to ensure all questions appeared as desired and that the survey functioned as planned before the initial release to the first cohort.

### Statistical Analysis

ProQOL analysis was performed according to the protocol provided in the 2009 version of the ProQOL manual, using mean scores of all participants for each category.^10^ Each question has 5 possible responses ranging from “Never” to “Very Often,” which corresponds to scores of 1–5 on a Likert scale. Per the manual, specific ProQOL questions pertain to burnout, compassion satisfaction, or secondary trauma (a sum score of ≤22 is considered “low,” 23–41 is “average,” and ≥42 is “high” for each variable).^10^ Qualitative data from the dedicated questionnaire were analyzed by common themes we identified as we reviewed the results which we analyzed using the “Stats IQ” tool on Qualtrics. The statistical significance threshold was set at *p* < 0.05. The strength of associations between nominal variables was measured with Cramér's V (values range from 0 reflecting no association to 1 reflecting a perfect association). The n value for each section was adjusted to account for nonresponse error for each component of the survey.

### Standard Protocol Approval, Registrations, and Participant Consents

This study was approved by the Institutional Review Board. Survey participation was voluntary and agreeing to participate implied voluntary consent. We adhered to the Checklist for Reporting Survey Studies.^15^

### Data Availability

Anonymized data not included within this report may be shared on reasonable request from any qualified investigator.

## Results

Of 195 third-year medical students comprising 6 cohorts completing their clerkship at different times during the 2023–2024 academic year who were invited to participate in the study, 100 provided responses (51.3% response rate). Of the respondents, 64% were from the institution's main campus, and 36% were from the 3 satellite campuses. In total, 81% of participants identified as White, with the remainder of the group identifying as Black/African American (6%), Asian (6%), American Arab/Middle Eastern/North African (2%), or preferred not to say (2%); 60% identified as female, 37% identified as male, and the remainder of respondents preferred not to say or did not answer this question.

Thirteen participants did not complete the ProQOL 5 portion of the survey. Based on the ProQOL 5 scoring criteria (n = 87), the mean score for compassion satisfaction (40.3 ± 5.9) was at the average level, and burnout levels were low (21.9 ± 5.4). Secondary traumatic stress levels (20.2 ± 4.9) were also low.

**Figure 1:**
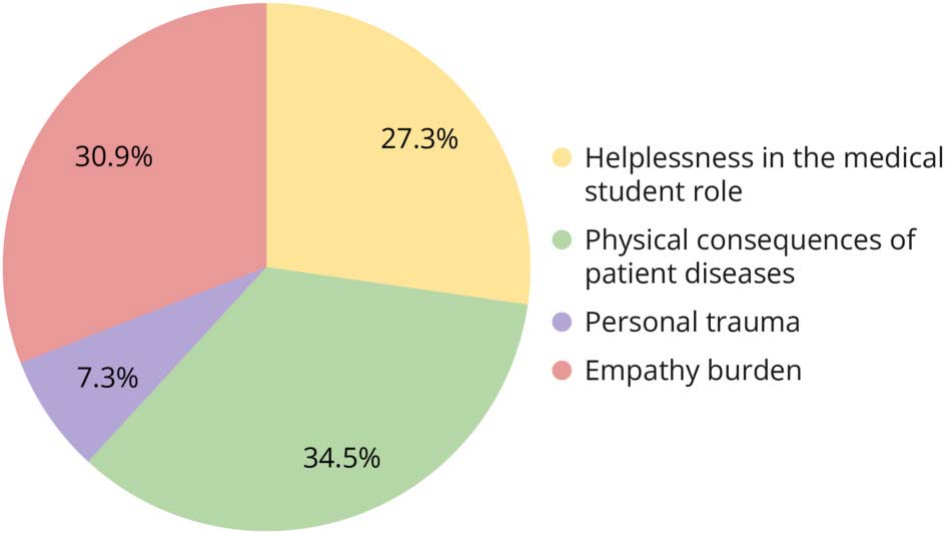
Themes of Emotional Burden Found in Medical Students Common themes of emotional burden identified from open response answers to the question, “What aspects do you think made emotionally distressing cases difficult for you?”

Emotional exhaustion was reported by 11.3% of survey respondents. By reading individual open response answers, we discerned 4 themes of emotional burden by identifying common phrases (Figure 1). Emotional burden was reported to be due to witnessing the physical consequences of the diseases affecting their patients by 34.5% of respondents. Empathy burden, in which students reflected on being overcome with empathy for patients and their families, was reported as the cause of emotional burden for 30.9% of these students. Feeling helpless in their role as a medical student was reported to have led to emotional burden in 27.3%, with many describing how they wished they could have had a more direct role in patient care. Finally, 7.3% of respondents reported that a history of personal trauma contributed to their emotional burden during the clerkship. Although many students reflected broadly on the emotions they felt when witnessing the effects of patients' conditions, there were neurology-specific responses that represented these themes. Respondents reported emotional exhaustion due to witnessing death of a stroke patient who had initially improved neurologically or realizing that their patients would likely not return to baseline as a result of massive strokes. Others identified how interacting with child abuse victims or having a personal history of trauma with intracranial bleeds increased their emotional exhaustion during the neurology portion of the clerkship.

**Figure 2:**
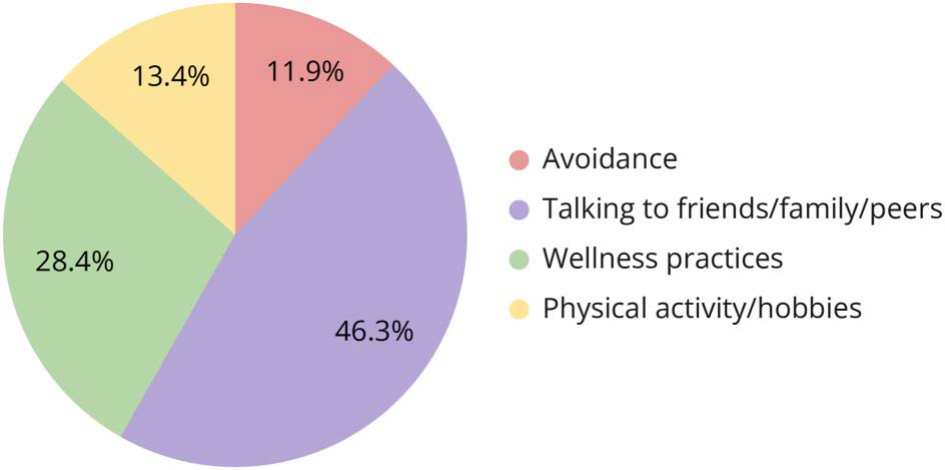
Categories of the Most Common Coping Strategies Utilized in Third-Year Medical Students Common coping skills used based on responses to the question “What specific coping strategies have you used to cope with emotionally distressing cases?”

**Figure 3:**
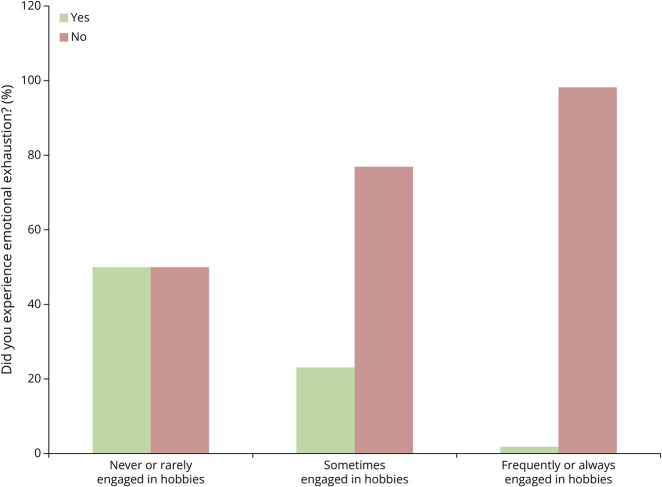
Hobbies/Activities Impact on Emotional Exhaustion Students' responses to the questions “Did you experience emotional exhaustion?” and “To what extent did you engage in enjoyable hobbies or activities during this rotation” were analyzed together (Cramér's V = 0.372; *p* = 0.001).

Students reported that they used 4 common coping strategies during the clerkship (Figure 2). The most common was talking with a support person, with 46.3% of students responding that they spoke to friends, family, and peers throughout the rotation. Wellness practices such as journaling, religious habits, and breathing exercises were used by 28.4% of respondents. In addition, 13.4% of students indicated that they participated in physical activity and other hobbies to help them cope during the rotation. Many respondents did not specify which type of hobbies they participated in, whereas some listed physical activity as their hobby. As a result, we combined these responses into one type of coping strategy (i.e., hobbies). Most of those who “sometimes” (95% CI 58.9%–86.2%) or “frequently or always” (95% CI 91%–99.7%) engaged in physical activities and other hobbies reported no emotional exhaustion compared with those who never engaged in these activities (Cramér's V = 0.372; *p* = 0.001; Figure 3); 11.9% of students reported that they coped by avoiding the struggles they were facing during the clerkship.

**Figure 4:**
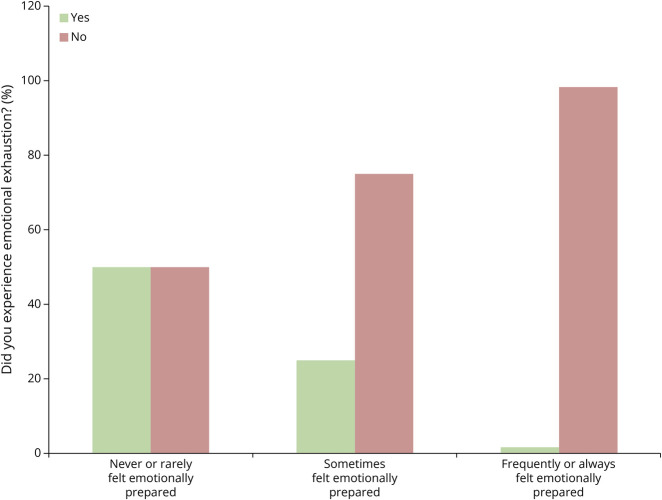
Emotional Preparedness to Handle Distressing Cases vs Emotional Exhaustion Students' responses to the questions “Did you experience emotional exhaustion?” and “To what extent did you feel emotionally prepared to handle distressing cases on rotation?” were analyzed together (Cramér's V = 0.395; *p* = 0.005).

**Figure 5:**
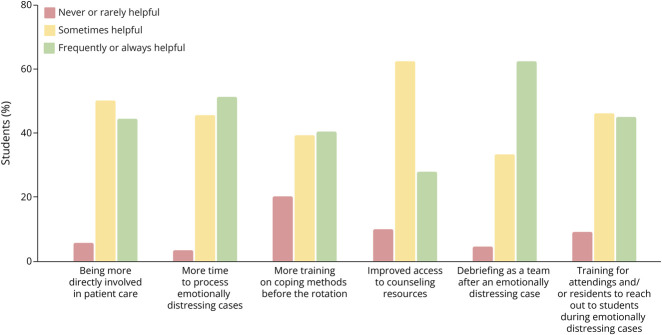
Student Input on Potential Curricular Changes Responses to the question, “Do you believe any of the following strategies would be helpful for students encountering emotionally distressing cases on rotations?”

Answers to the question, “In terms of the emotional burden, was this rotation what you expected?” revealed that 83.72% of participants expected the level of emotional burden they experienced. Most of those who “sometimes” (95% CI 61.7%–87.4%) or “frequently or always” (95% CI 90.6%–99.7%) felt prepared to handle emotionally distressing patients reported no emotional exhaustion (Cramér's V = 0.395; *p* = 0.005; Figure 4). Students were also asked to provide suggestions regarding potential strategies faculty could implement to prepare students to encounter emotionally distressing patients (Figure 5). The majority thought that the following strategies would sometimes or always be helpful: being more directly involved in patient care (93.83%), having more time to process emotionally distressing cases (96.30%), receiving more training on coping methods before the rotation (78.75%), having improved access to counseling resources (90.12%), debriefing as a team after an emotionally distressing case (95.06%), and providing training for attendings and/or residents to reach out to students during emotionally distressing situations (90.0%).

## Discussion

There are high rates of secondary trauma symptoms of emotional burden and burnout in neurology residents, fellows, and attendings.^1,2,5^ We found that medical students are also at risk for developing these symptoms after 6 weeks of exposure to a combined neurology (2 weeks)/emergency medicine (4 weeks) clerkship with 11.3% reporting emotional exhaustion, indicating that secondary trauma symptoms can be identified even before residency. Given that no previous studies assessed these issues during specific medical school clerkships, our findings, particularly themes of emotional exhaustion, effective coping mechanisms, and suggestions for curriculum changes, provide the opportunity to target specific areas to mitigate secondary trauma and its associated symptoms in the combined neurology/emergency medicine clerkship.

Interventions are available that can reduce the symptoms of secondary trauma in healthcare providers in later stages of their medical careers. Many apply to the themes for contributors of emotional exhaustion identified in our study population. For example, we found that a history of personal trauma was a key contributor to emotional exhaustion in respondents. One approach to address this is using mental health resources because prior exposure to trauma increases the risk of secondary traumatization.^8^ Self-compassion, using emotional intelligence, and having psychological flexibility are also beneficial in decreasing secondary trauma.^8^ Medical schools should provide students easy access to counseling resources and encourage their use as they navigate through their clinical years of training. It would be beneficial to provide students with protected time to attend therapy sessions during rotations to afford adequate opportunities to explore how any personal trauma may play a role in their experiences with challenging patients.

Although being unable to mitigate pain and suffering can be frustrating, the challenge of having students more involved with patient care, and presumably better able to encounter and react healthily to emotional risk, remains complex. One study found that establishing a compassionate connection with patients and their families led to increased compassion satisfaction for the provider, even when there were no other measures that could be taken to improve the underlying clinical situation.^9^ Medical students often have more time compared with other members of the care team to spend with their patients. Encouraging and teaching students how to effectively communicate with patients and their loved ones during distressing times can help them better connect with their patients, contribute more to their care, and as a result, help reduce compassion fatigue. In addition, there are neurologic assessment tools that medical students can easily be trained to administer, such as the Montreal Cognitive Assessment, National Institutes of Health Strokes Scale, and Denver Developmental Assessments. Although learning these techniques would not prevent students from being emotionally impacted by exposure to distressing patients, this would allow them to have active participation in patient care, decreasing the issue of feeling helpless in the student role. Interacting with patients in these ways could also improve student-patient connection, which can alleviate symptoms of secondary trauma.^9^

Career satisfaction is lower in those who reported burnout and is higher in trainees who have a better work-life balance and a supportive work environment.^5^ Similarly, our study revealed that engaging in enjoyable hobbies, including exercise, was associated with less emotional exhaustion. Third-year medical students must navigate a near full-time work schedule and find adequate time to study.^16^ Medical schools should encourage students to pursue hobbies and extracurricular activities and provide a supportive environment in which they feel empowered to do so to combat emotional exhaustion.

The most common theme contributing to emotional exhaustion in our study was witnessing the physical consequences of the diseases affecting their patients for the first time. During the neurology clerkship, these students work with residents and attendings who are involved in the care of distressing patients nearly every day, and as a result, what may be life-changing for a student to witness may be common for more experienced members of the team. Students in our survey showed a clear favorability for the medical team to debrief together after a distressing situation, with over 60% reporting it to be “frequently or always helpful.” Student schedules are also often much different than faculty while on these rotations, as they often move to various locations throughout the day such as from the inpatient setting in the morning to clinic in the afternoon. It would be beneficial for course directors to implement a protocol for faculty to check-in with students several times throughout the clerkship to provide them with a chance to debrief while also working with their varying schedules. Students often feel the pressure of high expectations. As a result, it is important for faculty to reach out to students for debriefing sessions to create a safe environment for them to discuss distressing experiences.

Third-year students are required to rotate in selected specialties, even if they do not intend to pursue them as a career. Some would not voluntarily choose a specialty in which most patients have distressing, emergent, or traumatic conditions, whereas others hope to spend most of their careers caring for such patients. In our study, over 80% of respondents indicated that they did expect the level of emotional burden they experienced in the neurology/emergency medicine clerkship with 98.3% who “frequently or always felt prepared” responding that did not experience emotional exhaustion. For those who did not feel prepared, however, the responsibility of improving preparation may fall at the individual or institutional level. Medical schools should attempt to optimally prepare students for what to expect related to emotional distress before the rotations. This is especially true for the neurology clerkship, in which students often have their first encounter with performing physical examinations on patients who are nonverbal or not interactive.

Our study has several limitations. The sample was limited (n = 100) and came from a single institution. Studies in other medical schools could help determine whether the extent of secondary trauma varies across settings. The ProQOL portion of the survey was not completed by 13 students. We suspect this was due to the longer length of this section compared with the rest of the survey. In addition, as the themes of emotional exhaustion were identified based on responses to open-ended questions, future qualitative studies on these themes would be useful as studies show that open-response answers may not produce strong stand-alone insights.^17^ Furthermore, the ProQOL survey asks participants to reflect back over the previous 30 days when answering the questions. Our data reflect student experiences from the combined 6-week neurology/emergency medicine clerkship, and we could not determine whether responses differentially reflected the 2 portions of the rotation. Witnessing effects of child abuse, intracranial hemorrhages, and strokes during the neurology component of the clerkship, however, were noted as some of the causes of emotional exhaustion in open-response answers. As a result, follow-up studies are needed to determine the specific impacts on the emergency medicine and neurology components of this clerkship. We did not determine whether the extent of reported secondary trauma may have changed over the course of the academic year. Students who had this clerkship earlier in the year may have greater emotional exhaustion due to having less clinical experience compared to later groups, or students rotating through the clerkship at the end of the year may have been more burned out and thus experienced more emotional exhaustion. Additional analyses with a larger sample size could compare experiences based on when during the course of the curriculum the students rotated through the clerkship. Given the lack of research on this topic in medical students, it is unknown if these issues are seen in other specific clerkships. Future studies are warranted to determine if similar findings are present after students rotate with other specialties.

Our study highlights that symptoms of secondary trauma can develop in medical students after a combined neurology/emergency medicine clerkship. Strategies to alleviate secondary traumatization should be implemented during medical school to address key contributors to emotional exhaustion and help prepare students for resilience during residency and throughout their careers. Future studies are warranted to determine additional areas in which secondary trauma prevention can be implemented in medical education.

## Glossary

ProQOL: Professional Quality of Life
